# Socializing a group of male Asian elephants in a semi-captive facility in Lao PDR

**DOI:** 10.1371/journal.pone.0332944

**Published:** 2025-11-26

**Authors:** Ana Belén López Pérez, Janine L. Brown, Shifra Z. Goldenberg, Amapola Rey, Jaruwan Khonmee

**Affiliations:** 1 Faculty of Veterinary Medicine, Chiang Mai University, Chiang Mai, Thailand; 2 Elephant, Wildlife and Companion Animals Research Group, Chiang Mai University, Chiang Mai, Thailand; 3 Elephant Conservation Center, Sayaboury, Lao PDR; 4 Smithsonian National Zoo & Conservation Biology Institute, Center for Species Survival, Front Royal, Virginia, United States of America; 5 Conservation Science and Wildlife Health, San Diego Zoo Wildlife Alliance, Escondido, California, United States of America; Universite de Liege, BELGIUM

## Abstract

This study documents the introduction process of eight unrelated captive male Asian elephants in a free-contact management setting in Laos, utilizing a two-phase introduction process comprising limited tactile contact followed by full physical introduction. Behavioral data were collected using all-occurrence and focal-animal sampling, while fecal samples were analyzed for glucocorticoid (fGCM) and androgen (fAM) metabolite concentrations to assess physiological responses. Results indicated a prevalence of affiliative over aggressive or submissive behaviors throughout both introduction phases, supporting the idea that unrelated males can create social bonds without excessive aggression in captive settings. Affiliative behaviors declined over time during the limited tactile contact phase, then stabilized during physical introductions. Aggressive and submissive behaviors were consistently low throughout the study. Individual variations in behavior were observed, highlighting the importance of considering temperament in elephant introductions. No significant differences were found in fGCM concentrations before or after social introductions among the males, suggesting that the process did not cause substantial physiological stress. Only one male exhibited decreased fAM concentrations after social interactions, which could indicate testosterone suppression from more dominant males. During the limited contact period, aggressive interactions were positively associated with fGCM concentrations, whereas a longer duration since first introduction was associated with a decrease. In the physical introduction step, the number of days since the first introduction positively predicted an increase in fGCM concentrations, while the fAM concentration before social interactions negatively predicted fGCM. In addition, age was significantly positively predictive of fAM concentrations. These findings challenge traditional views on male elephant sociality under captive conditions and suggest that, with proper management, forming all-male groups can be a viable option for conservation and *ex situ* management programs. This study emphasizes the importance of gradual introduction processes, individual monitoring, and long-term behavioral observations in the successful introduction of unrelated captive male Asian elephants.

## Introduction

Asian elephant (*Elephas maximus*) population numbers continue to decline and have been reduced by at least 50% across all 13 range countries in the last three generations [1]. Of the estimated 35,000–50,000 extant Asian elephants, approximately 15,000 live under human care, participating in tourism, logging, and religious activities, with fewer than 2,000 in zoos [2]. If managed appropriately, captive populations, especially in range countries, can play a vital role in conservation through captive breeding for purposes such as reintroduction, public education, and scientific research. Given the critical state of Asian elephant populations and the significant number of individuals living under human care, it is crucial to implement effective conservation and management strategies. One key aspect is understanding elephant behavior and social dynamics in the wild, the lack of which could be detrimental to the proper management of captive counterparts [3,4]. Bridging insights from wild elephant behavior to captive management is crucial to ensure that captive populations effectively support elephant conservation.

Consideration of species-typical social behavior in captive settings has improved welfare across taxa. In cotton-top tamarins *(Saguinus oedipus*), resemblance to wild-type groups led to increased breeding success, higher infant survival, and lower incidences of abortion, stillbirths, and parental neglect [5]. In male baboons (*Papio anubis*), social integration was associated with lower basal cortisol levels, suggesting reduced stress in socially integrated individuals [6]. Providing chimpanzees (*Pan troglodytes*) with the opportunity to engage in fission-fusion dynamics, akin to those found in wild populations, led to lower rates of aggression [7]. Finally, Schapiro (2002) [8] found that species-typical behavior in rhesus macaques (Macaca mulatta) was increased, and abnormal behavior was decreased by social rather than single housing. In Asian elephants, animals managed in social groups show lower rates of stereotypic behavior than those housed in isolation [9]. Additionally, herd stability significantly influenced the survival of calves among both Asian and African savanna elephants (Loxodonta africana) in European zoos [10]. Recent research has further illuminated the complex social behaviors and relationships that elephants exhibit, even in captive settings. For instance, Williams et al. [3] found that social interactions among zoo-housed elephants were influenced by factors such as age, personality, and the presence of calves within the herd, not unlike those in the wild. Moreover, Asian elephant females can maintain strong friendships even after long separations [11], underscoring the importance of considering social dynamics in captive management.

While the importance of female sociality is well-established, there is a growing recognition that male elephant welfare can also be enhanced by managing them socially, not only with females but also with other males. Although elephant males were historically considered primarily solitary [12,13], the richness and complexity of male-male social relationships are now widely acknowledged. For example, male elephants in Kenya were observed in all-male groups 46% of the time [14], while in Botswana, older males were shown to play a central role in male society, particularly in guiding younger, inexperienced males to critical resources [15]. Among Asian elephants, a study conducted in southern India concluded that male elephants spent 43% of their time in mixed-sex groups, 34% alone, and 23% in all-male groups ranging from 2 to 12 individuals [16]. In Sri Lanka, male elephants congregate in small groups of up to seven animals, with approximately 20% of elephant sightings comprising all-male groups [17]. These findings support the idea that adult males alternate time spent alone, in mixed-sex groups, and in male groups, albeit with weaker social bonds than those of females [18–20].

The amount of time that wild males spend alone or with conspecifics depends on various factors, including age and reproductive state [18,19,21,22], forage abundance, kin relationships [14], and human activities [16]. Consequently, housing male elephants singly in captivity, with limited to no opportunity to interact with other elephants, fails to provide the social interactions they would naturally seek in the wild—interactions that are key to their development and social competence, potentially compromising their well-being. Recognizing this, western zoos have begun integrating males into female groups or creating bachelor groups [23–26]. However, in range countries, males are still primarily housed individually due to concerns about aggression, particularly during musth periods [27]. Furthermore, regional guidelines for elephant care and husbandry often overlook the social needs of male elephants [28–30], and there is a lack of clear guidance on introducing unrelated male elephants into all-male groups or female herds. Various approaches have been used to introduce unrelated animals, ranging from immediate co-housing [31] to a gradual introduction through sensory and limited tactile contact before sharing physical space [26,32,33]. While introductions can succeed without rigorous protocols, Burks et al. [23] found that a systematic method—progressing from sensory access to restricted physical access and finally unrestricted physical access—effectively managed aggression during female African elephant introductions.

Introductions of new individuals can be stressful; therefore, glucocorticoid concentrations are commonly used as stress indicators for welfare management [34]. Glucocorticoid measures have also been used to monitor social introductions in zoo female elephants [35,23]. In addition, the initial months of an introduction are crucial for establishing social hierarchies through dominance interactions (López Pérez et al. in prep), which may be influenced by testosterone. Testosterone has been proposed to facilitate socioemotional mechanisms that enhance an individual’s ability to achieve and maintain social dominance [36]. Furthermore, across the animal kingdom, testosterone concentrations fluctuate rapidly in the context of competitive interactions, with winners demonstrating increased testosterone concentrations relative to losers [37]. Therefore, testosterone may mediate social interactions of male elephants during introduction periods.

The goal of this study was to assess the integration of eight unrelated males at the Elephant Conservation Center (ECC) in Lao PDR by combining measures of behavior and endocrine responses as guides. Specifically, we investigated whether 1) social behaviors (affiliative, aggressive, and submissive) change as a function of time, and 2) how fecal glucocorticoid (fGCM) and fecal androgen (fAM) metabolite concentrations were related to any changes in male-male social interactions. Understanding how the introduction process affects behavior and physiological function can provide valuable insights to improve management standards for and enhance the welfare of captive male elephants, potentially contributing to conservation efforts and the recovery of this endangered species.

## Materials and methods

### Animal ethical consent

This study was approved by the Institutional Animal Care and Use Committee, Faculty of Veterinary Medicine, Chiang Mai University, Chiang Mai, Thailand (FVM-ACUC, permit number (S5/2564).

### Study animals

The study was conducted at the Elephant Conservation Center (ECC) in Sayaboury province, Laos PDR (Latitude, 19°14’0.29“N; Longitude, 101°39’12.06”E; Elevation 301.752 m – 393.192 m) from February 2020 to June 2022. The ECC was founded in 2011 and, at the time of the study, was home to 30 elephants (20 females and 10 males). The ECC serves dual purposes: as an ecotourism destination and a conservation center dedicated to preserving Lao elephants through established research, breeding, and reintroduction programs. Of the elephants at the ECC, 18 were owned by the center, one was rented, and 11 were confiscated by the government and placed under ECC care. All elephants resided within a 530-hectare forest concession year-round, with access to specific areas changing daily. During the day, movements were based on several factors, including food availability, social interactions, health status, and seasonal conditions. At night, all elephants were restrained with 30–40 m chains in different locations throughout the forest. While elephants had limited access to certain parts of the forest on any given day, over the course of the year, they had access to the entire 530-hectare area.

Eight male Asian elephants (age range: 9–60 years at study start) were the subjects of this study (Table 1). These males were captive-born in Laos and privately owned by locals before being obtained by the ECC. Seven were separated from their mothers at the age of 3 years, as is typical for captive elephants in Laos, and grew up with little to no opportunity to freely socialize with conspecifics. Only the youngest male, Suriya—who arrived at the ECC with his mother when he was less than a year old—was not separated and grew up in a female herd, having daily social interactions with other elephants. At the ECC, males are handled by mahouts (i.e., keepers) in a traditional free-contact management system [38]. Before physical introductions, some males had limited direct contact with other males during weekly walks (2–3 times/week) or temporary social interactions with females for breeding purposes. Otherwise, males were alone foraging in the forest. Males in musth were isolated from other elephants and tethered in the forest using a 20–25 m chain within reach of natural food and water until it was safe to reintegrate them.

**Table 1:** Male elephant subjects included in the study.

Subject	Year of birth	Previous job	Social background before arriving to the ECC	Arrival at the ECC	Social background at the ECC
Thong Khoun (TK)	1960	Logging	Little to no opportunity to freely socialize with conspecifics.	2018	Limited physical interactions with BP, XY, PKS, and DKS during walks. Visual, auditory, and olfactory contact with BB, S, and JB. Died the 24th of April 2022. Temporary encounters with female elephants for breeding purposes.
Xaiyo (XY)	1984	Logging	Little to no opportunity to freely socialize with conspecifics.	2018	Limited physical interactions with BP, XY, PKS, and DKS during walks. Visual, auditory, and olfactory contact with BB, S, and JB. Temporary encounters with female elephants for breeding purposes. Left the ECC the 8th of May 2021.
Bua Ban (BB)	1970	Logging	Little to no opportunity to freely socialize with conspecifics.	2017	Visual, auditory, and olfactory contact with all the males included in the study. Temporary encounters with female elephants for breeding purposes.
Joun Ban (JB)	1987	Logging/ tourism	Little to no opportunity to freely socialize with conspecifics. Daily contact through a barrier with a female elephant from 2017 to 2019.	2019	Visual, auditory, and olfactory contact with all the males included in the study. Temporary encounters with female elephants for breeding purposes.
Boun Pheng (BP)	1992	Logging	Little to no opportunity to freely socialize with conspecifics.	2018	Limited physical interactions with TK, XY, PKS, and DKS during walks. Visual, auditory, and olfactory contact with BB, S, JB. Temporary encounters with female elephants for breeding purposes.
Pai Kham Sing (PKS)	2001	Logging	Little to no opportunity to freely socialize with conspecifics.	2018	Limited physical interactions with TK, XY, BP, and DKS during walks. Visual, auditory, and olfactory contact with BB, S, and JB. Temporary encounters with female elephants for breeding purposes.
Do Kham Son (DKS)	2003	Circus shows	Little to no opportunity to freely socialize with conspecifics.	2018	Limited physical interactions with TK, XY, BP, and PKS during walks. Visual, auditory, and olfactory contact with BB, S, and JB.
Suriya (S)	2011	NA	Daily social interactions with females.	2011	Visual, auditory, and olfactory contact with all the males included in the study. Daily interaction with female herd.

### Introduction phases and behavioral data collection

Burks et al. [23] described three sequential phases to introduce unrelated captive elephants and effectively manage potential aggression: visual contact (consisting of visual, auditory, and olfactory contact with conspecifics); limited tactile contact (individuals were allowed limited contact through separating adjacent stalls or yards); and physical introduction (individuals were placed in the same physical space with no barriers). In this study, all males had visual, auditory, and olfactory contact with each other for at least 1 year by sharing feeding and bathing grounds during daily routines. For that reason, the introduction protocol at ECC was reduced to the last two phases described by Burks et al. [23]: limited tactile contact and physical introduction (Table 2). At the beginning of the limited contact step, mahouts allowed male elephants to interact for only short periods (10–15 minutes) due to concerns about potential aggressive interactions. Because it was not feasible to conduct focal sampling during these times, all-occurrence sampling was used [39] to collect behavioral data from all males. During the physical contact step, elephants were placed together for longer periods (up to 3 hours), making all-occurrence sampling impractical; therefore, behavioral data collection switched to focal sampling [39]. Since all-occurrence sampling is a focal animal sampling technique for an entire group at once, the behaviors collected should be comparable between the two methods [39].

**Table 2:** Behavioral data collection summary.

Introduction type	Time period	Data collection type	Observations	Total hours observed
Limited tactile contact	13-Feb-2020 to 9-Jul-2020	All occurrence sampling	83	34
Physical introduction	15-Jul-2020 to 16-Jun-2022	Focal-animal sampling	1107	351

Introductions were not attempted if the weather was extreme (i.e., heavy rain, agricultural fires), elephants were sick and/or needed to rest, or males were in musth.

### Limited tactile contact

The ECC does not have barriers or fences strong enough to stop aggression between males, so the limited tactile contact phase was accomplished through walks around the ECC concession (S1 Fig), which occurred over 3 consecutive days per week, lasting 2–4 hours, depending on weather conditions and elephant behavior. During these walks, males walked in a single-file line with mahouts riding on their necks. This arrangement allowed the elephants to become familiar with each other in a controlled manner and mahouts to manage any potential aggression. The order of elephants in the line was varied daily to ensure all males had opportunities to interact throughout this phase.

During walks, elephants and mahouts took periodic breaks in open, flat areas, which allowed elephants to freely interact with each other while mahouts remained nearby to supervise behavior. After each introduction session, the collected data were reviewed by the observer and mahouts, specifically, rates of aggressive and affiliative behaviors exhibited by the group, as well as the context in which aggression occurred. If, for 3 consecutive days, the rate of affiliative behaviors exceeded the rate of aggressive behaviors, and any aggression observed did not require intervention by the mahouts (i.e., was not considered a risk to the elephant or mahout), the introduction process was progressed the following week. This progression consisted of increasing the duration of walk breaks and allowing a greater distance between the mahouts and elephants during rest periods. Initially, the breaks were short (10–15 minutes) but gradually increased to 90 minutes over a period of 104 days. When 90-minute breaks were reached and the rate of aggressive behaviors remained consistently low, without requiring intervention, the introduction was moved to the physical introduction step.

Based on protocols developed for this study following Burks et al. [23], dyads exhibiting high rates of affiliative behaviors and low rates of aggression were considered suitable for physical introduction. By contrast, dyads showing high rates of aggression and low rates of affiliative behavior would be further evaluated. If the majority of aggressive behaviors within the dyad were non-contact aggressive behaviors (i.e., did not involve physical confrontation), the pair would be considered suitable to move to the physical contact phase. However, if the majority were contact-aggression, those individuals would not be introduced until aggressive rates were reduced. In this study, no dyads were deemed unsuitable, allowing us to proceed with introducing all eight males together during the physical contact phase.

### Physical introduction

During the physical introduction phase, all males were placed together 3 consecutive days per week for 1.5–3 hours (between 0900 and 1200 hours) in large patches of open forest (3–11 ha). The mahouts selected the areas based on the amount of natural food, shade, and water points. When food, shelter, and water became scarce, mahouts selected a new location to minimize male competition. Once the area was chosen, males were released, allowing them to freely interact with other males or remain alone.

Mahouts remained at least 30 m from the elephants during observations. They intervened only if elephants moved too far away (>150–200 m) or were involved in dangerous situations that needed immediate intervention, such as approaching agricultural areas or engaging in aggressive fights.

### Data collection

An ethogram was developed using descriptions of social behaviors drawn from prior studies (Table 3) [3,23,40–42]. The selected behaviors were then grouped into categories considered functionally similar [26,23,43–45] and were defined as aggressive, affiliative, submissive, and other. The aggressive category was subdivided into aggressive contact and aggressive non-contact as previously described [26,23,44,45]. Data on social behavior was collected from each male between 0900 and 1200 hours, 3 consecutive days per week throughout the study period (February 2020 to June 2022). Elephants were observed for at least 90 minutes each day from a minimum distance of 30 m so that the observer would not interfere with their behavior. The lead author collected all behavioral observation data. Throughout the limited tactile contact period, all occurrence sampling [39] recorded behaviors during breaks until the group resumed walking. For safety reasons, only non-musth males were present during walks. The start and end times, the initiator/receiver of each interaction, the individuals present in the group during the observation, and the temperature were also recorded.

**Table 3:** Elephant ethogram grouped by behavioral category.

Behavior category		Behaviors	Definition
Aggressive	Contact	Push	Initiator uses head to forceful push the recipient elephant, resulting in the target elephant moving. Pushes during play may be less intense and followed by affiliative behaviors.
		Drive	Initiator uses head, tusks or trunk to push the rear of another elephant, maintaining contact while both elephants move at least one body length. Drives during play may be less intense and followed by affiliative behaviors.
		Kick	Initiator strikes at recipient with rear front foot or rear limb. Kicks during play may be less intense and followed by affiliative behaviors.
		Spar dominant	Initiator engages in a strong head-to-head contact, pushing trunks, tusking, shoving, wrestling or trunk entwining with another elephant.
		Trunk over back	Initiator places at least two-thirds of his trunk over the recipient back.
		Trunk over head	Initiator places the trunk or head on top of the recipient elephant head or neck.
	Non-contact	Approach head high	Initiator moves toward recipient with head above shoulders and ears extended.
		Ears extended	Initiator fully extends his ears while facing another elephant.
		Charge	Initiator rapidly approaches another elephant with a rapid gait towards a stationary conspecific without making contact.
		Chase	Initiator rapidly pursues the receiver who is moving away from the initiator.
		Displace	Initiator approaches the recipient who moves away from his position without physical contact.
Affiliative	Spar play (escalated spar play)		Initiator pushes trunks, tusks, shoves, wrestles with recipient elephant. This is associated with relaxed contact including trunks entwined, trunk touching to each other’s bodies, especially to mouths, head low, and ears drooped.
	Head butt play		Initiator pushes the butt of the recipient. The recipient responded with an affiliative behavior.
	Push play		Initiator pushes head-to-head, head-to-body or body-to-body of the recipient with another elephant in play.
	Drive play		Initiator uses head, tusks or trunk to push the rear of another elephant, maintaining contact while both elephants move at least one body length. The recipient responded with an affiliative behavior.
	Kneel play		Initiator kneel on the forelegs or rear legs in response to affiliative behaviors of another elephant.
	Palatal pit area		Initiator uses trunk to touch or to place the tip of the trunk inside the recipient elephant’s mouth.
	Anal touch		Initiator uses trunk to touch recipient elephant’s anus.
	Genital touch		Initiator uses trunk to touch another elephant’s genitals.
	Non-aggressive approach		Initiator calmly moves toward the recipient with head low and flapping ears.
	Follow		Initiator walks calmly behind another elephant withing a few body lengths.
	Body touch		Initiator uses head or body to contact the recipient elephant.
	Trunk touch		Initiator touches with trunk to another elephant’s head, trunk, body, front legs, or mammary glands.
	Lie down play		Initiator lay on one side while interacting with the recipient elephant in affiliative context.
Submissive	Scream		Initiator performs a high frequency vocalization emitted from open mouth.
	Back up		Initiator walks backward into the recipient elephant.
	Back away		Initiator walks backward away from recipient elephant while facing recipient.
	Present back		Initiator presents their back when the recipient elephant approaches.
	Run away		Initiator flees from recipient elephant in response to aggressive contact, display, or approach.
	Blow air		Initiator expels air audibly from his trunk.
	Trunk ground		Initiator hits the ground with the trunk making an audible noise while blowing air.
	Trumpet		Loud, high-pitched, resonant vocalization.
Other	Sniff		Initiator extends trunk and sniffs toward the recipient elephant without touching.
	Stereotypy		Initiator performs stereotypic behavior, including head-bobbing or pacing.
	Other		Any other behavior not described in the ethogram. Describe the behavior and classify it into one of the categories: other aggressive contact, other aggressive non-contact, other submissive, and other affiliative.

During physical introductions, 20-minute focal-animal sampling [39] was used to assess male behavior using the same ethogram as above. For safety reasons, only non-musth males were placed with other males during this period. Start and end times, the initiator/receiver of each interaction, the individuals present in the group during the observation, and the temperature were also recorded [23].

### Fecal sample collection

Fresh fecal samples were collected between 0800 and 1200 hours before social interactions and then 48 hours after the 3 consecutive days of social interactions to account for fecal excretion lag times. Approximately 50 g of fecal material was collected from at least three parts of the central portion of the fecal bolus and placed in ziplock bags. Gloves were worn during collection to prevent sample contamination. The bags were labeled with the name of the elephant, collection time, and date, and were immediately placed in a Styrofoam cooler with ice. The samples were kept in a cooler filled with ice for 6–7 hours until transported to the ECC endocrine laboratory, where they were immediately frozen at −20ºC.

### Hormone processing and extraction

Fecal samples were extracted as described by Norkaew et al. [46]. Briefly, wet samples were dried in a conventional oven at 60ºC for ~48 hours. Dry samples were pulverized and sifted using a steel mesh strainer to remove fibrous material. The resulting fecal powder was stored at −20ºC until extraction. The frozen dried fecal powder was thawed at room temperature, mixed well, and 0.1 g (± 0.003) of powdered feces placed in a 16 x 135 mm glass tube. Samples were extracted in 90% ethanol by boiling in a water bath (95ºC) for 20 minutes and adding 100% ethanol as needed to keep from boiling dry. Samples were centrifuged at 1500 x g for 20 minutes. The extracts were decanted into a second set of tubes, 95% ethanol was added to the fecal pellets, and the tubes were centrifuged at 1500 x g for another 20 minutes. Extracts were pooled and then dried under air in a 50ºC water bath. Dried extracts were reconstituted in 1 ml assay buffer and stored at –20ºC until enzyme immunoassay (EIA) analysis [46].

### Hormone analyses

Concentrations of fGCM were determined using a double-antibody EIA with a polyclonal rabbit anti-corticosterone antibody (CJM006) validated for Asian elephants [47] and described by Norkaew et al. [46]. The absorbance was measured at 450 nm by a microplate reader (TECAN, Sunrise microplate reader, Salzburg, Austria). Assay sensitivity (based on 90% binding) was 0.14 ng/ml. Samples (25 μl) were diluted 1:6 or 1:8 in assay buffer for analysis in duplicates. The inter-assay CV for high (30% binding) and low (70% binding) control samples was < 15%. Samples were reanalyzed if the duplicate CV was > 10%; thus, intra-assay CVs were <10%.

Concentrations of fAM were quantified by a double-antibody EIA validated for Asian elephants [48] utilizing an anti-testosterone antibody (R156/7). Fecal extracts (25 μl) were diluted 1:16 in assay buffer, and absorbance was measured at 450 nm. Assay sensitivity (based on 90% binding) was 0.04 ng/ml, and the intra- and inter-assay CVs were <10% and <15%, respectively. Fecal data are expressed as ng/g dried feces.

### Statistical analysis

Analyses were based on 385 hours of behavioral observations and 1090 fecal samples (545 samples before social interactions and 545 samples 48 hours after social interactions) from the eight males during the two phases of the introduction process.

Rates of initiated behaviors per minute were calculated for each behavioral category and for each individual by dividing the total number of initiations by the total observation time. These rates were computed separately for the limited tactile contact and physical introduction phases. Rates of affiliative, aggressive, and submissive behavior were plotted against the number of days since the first introduction to visualize whether behavioral rates increased or decreased over time. A Spearman correlation test was used to determine if the observed trend over time was statistically significant.

A Wilcoxon signed-rank test was used to compare population-level fAM and fGCM concentrations before and after social interactions. Since social interactions occurred over 3 consecutive days, the counts of behaviors were calculated by summing all observed behaviors within each category during these 3-day periods. Similarly, the total observation time was calculated by summing the time the elephants were observed over these 3 days. Observation times were tallied separately for each individual male, as not all males participated in every possible interaction day, resulting in variable observation durations across individuals. Once behavior rates were calculated for each 3-day period, corresponding pre- and post-interaction fAM and fGCM concentrations were assigned to match the timing of each rate.

In addition to assessing whether all before-interaction fGCM and fAM values were significantly different from all after-interaction values as described above, we assessed differences at the individual pairwise level (i.e., whether a male’s endocrine values increased from their starting point before the interaction period). To determine whether male-male interactions influenced fAM and fGCM concentrations, we calculated the change in fAM and fGCM concentrations for each male after each social interaction period. This was done by subtracting the fAM and fGCM concentrations for a given time period (after minus before social interactions). Because these values were continuous and could be positive or negative, we then constructed Gaussian error distribution models with “after minus before” concentration differences as response variables in the glmmTMB package [49] using R Statistical Software v 4.1.2 [50]. The covariates considered for all models included days since first introduction, mean temperature over the 3 days, male age, counts of affiliative interactions, and counts of aggressive interactions. For the fGCM models, we also included fAM concentration before the interaction period as a covariate because elevated fAM in males is known to influence social interactions [51,52]. Variables were scaled prior to inclusion in models. Male ID was used as a random effect to account for individual variations and to avoid pseudo-replication. We conducted these models separately for the limited tactile contact and physical introduction phases and assessed models fit using the DHARMa package in R [53]. However, these models did not meet diagnostic criteria, so we converted the response variable “after minus before” values for fAM and fGCM into a binary variable to simplify the models (1 = fAM and fGCM increased within the male after an interaction period; 0 = fAM and fGCM decreased within the male following an interaction period). We then ran logistic regression models with these binary variables as the response variables for fAM and fGCM models, respectively, using the lme4 package in R and the same model features described above [54]. Diagnostic assessments for these models indicated good fit, and we therefore present results from logistic regression models.

## Results

**Fig 1:**
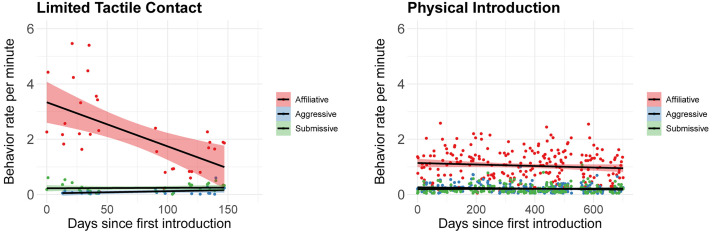
Overall temporal changes in behavioral rates during introduction phases. Scatter plots with trend lines showing the temporal progression of three behavioral categories (affiliative, aggressive, and submissive) during the limited tactile contact and the physical introduction.

Affiliative behaviors were the predominant form of interaction throughout both the limited tactile contact and physical introduction phases, consistently occurring at higher rates per minute than either aggressive or submissive behaviors over time (Fig 1). Analysis using the Spearman correlation test revealed a significant decrease in affiliative behavior rates over time during the limited tactile contact period (rho = −0.54, p = 0.0014), whereas affiliative behaviors remained relatively stable during the physical introduction phase, showing no significant temporal change (rho = −0.09, p = 0.19). By contrast, aggressive behaviors were notably absent during the initial days of the limited tactile contact phase, not appearing until day 13, albeit at low rates throughout, with no discernible trend over time (rho = 0.01, p = 0.97). In contrast, during the physical introduction period, aggressive behaviors began at similarly low rates but declined as time progressed (rho = −0.19, p = 0.004). Submissive behaviors were consistently low across both phases, with rates that remained unchanged over time, as reflected in non-significant correlations during both the limited tactile contact (rho = 0.08, p = 0.68) and physical introduction steps (rho = 0.02, p = 0.76).

**Fig 2:**
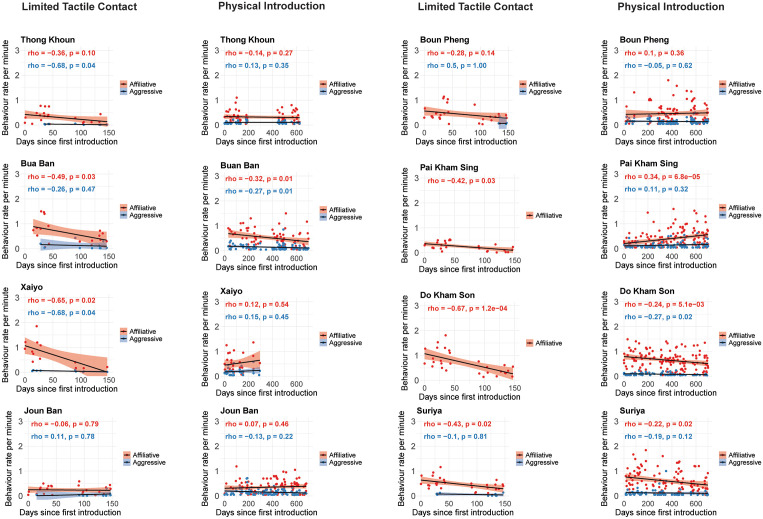
Temporal changes in behavioral rates during introduction phases per individual. Scatter plots with trend lines of each male in the study (n = 8) showing the temporal progression of two behavioral categories (affiliative and aggressive) during the limited tactile contact and the full physical introduction phases. The strength and significance of behavioral rate changes over time were assessed using Spearman’s rank correlation analyses.

Behavior rates plotted over time by individual elephant confirmed the trends described above (Fig 2). Notably, none of the males initiated aggressive behavior during the first days of the limited-contact phase, while two individuals (DKS and PKS) never initiated aggressive behaviors during the entire period. The main trend observed by four of the males (BB, DKS, S, and TK) was that affiliative behavior decreased over time, while aggressive behaviors were performed at low but consistent rates throughout the two introduction periods. However, the other four males (PKS, XY, BP, and JB) showed an increase in affiliation rate over time during the full physical introduction steps, but this trend was only significant for PKS.

**Fig 3:**
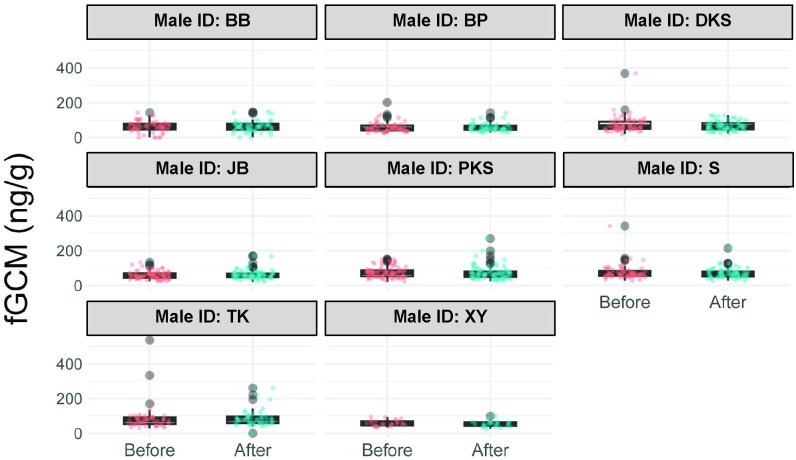
Fecal glucocorticoid metabolite (fGCM) concentrations before and 48 hours after social interactions for each male. Each plot shows the median (horizontal line), 25 and 75% quartile ranges, and minimum and maximum ranges (whiskers). Before = fecal samples collected before social interactions; After = fecal samples collected 48 hours after social interactions.

**Fig 4:**
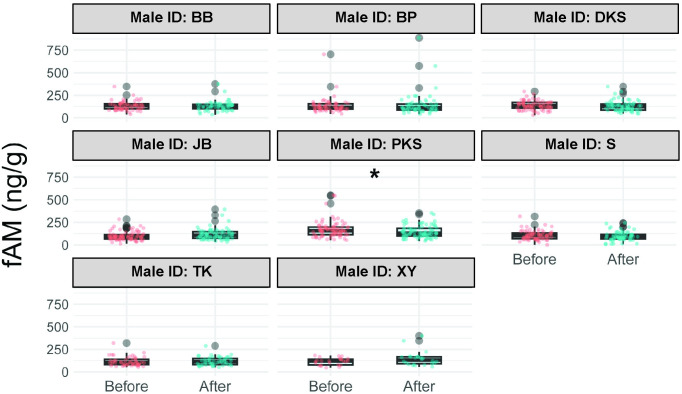
Fecal androgen metabolite (fAM) concentrations before and 48 hours after social interactions for each male. Each plot shows the median (horizontal line), 25 and 75% quartile ranges, and minimum and maximum ranges (whiskers). Before = fecal samples collected before social interactions; After = fecal samples collected 48 hours after social interactions. The * symbol denotes statistical significance (p < 0.05).

Considerable variability was observed in both fAM and fGCM concentrations across elephants in the study (S2 Table). Despite this high variability, changes in fGCM concentrations before and after interactions were not significant in any of the males (S3 Table, Fig 3). Similarly, changes in fAM concentrations before and after social interactions were not significant for all males with the exception of PKS, whose fAM decreased significantly after the social interactions (S4 Table, Fig 4).

**Fig 5:**
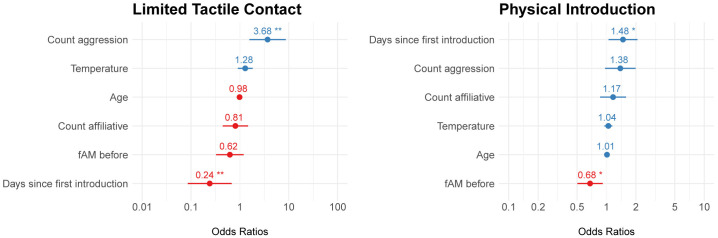
Odds ratios for covariates for fecal glucocorticoid metabolite (fGCM) logistic regression models. Blue color indicates positive effect, while red color indicates negative effect between the covariate and fGCM. (*) p-value<0.01; (**) p-value< 0.001.

The logistic regression model for differences in fGCM before and after introductions was a good fit for the data for the limited and full contact periods (S5 Fig). For the limited contact period model, the number of aggressive interactions during an interaction period was positively predictive of an increase in fGCM following the interaction period. Days since first introduction was negatively predictive of an increase in fGCM following the interaction period (Fig 5). No other covariates were significant. For the full contact period model, days since introduction was significantly positively predictive of an increase in fGCM, while fAM before the interaction period was significantly negatively predictive of an increase in fGCM.

**Fig 6:**
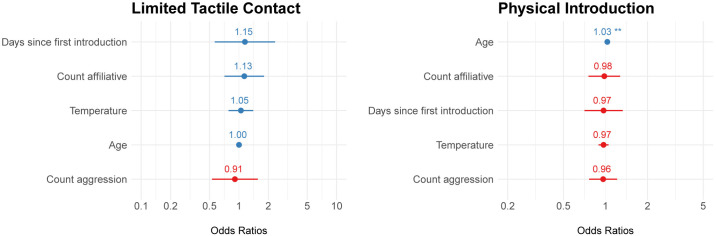
Odds ratios for covariates of the fecal androgen metabolite (fAM) logistic regression models. Blue color indicates a positive effect, while red color indicates a negative effect between the covariate and fAM. (*) p-value<0.01; (**) p-value< 0.001.

The logistic regression model for the difference in fAM before and after introductions was a good fit for the data for the limited and full contact periods (S6 Fig). For the limited contact period model, none of the covariates were significant (Fig 6). For the full contact period model, age was significantly positively predictive of an increase in fAM. The rest of the covariates were not significant.

## Discussion

This study documented, for the first time, how the integration and socialization of unrelated male Asian elephants in a free-contact management setting affected behavior and hormonal responses. In stark contrast to their reputation as being highly aggressive [55], this study revealed a consistent trend of higher rates of affiliative behavior per minute over aggressive or submissive behaviors over time during both the limited tactile contact and full physical introduction phases in all individuals, regardless of age. Affiliative behaviors gradually decreased over time during the limited tactile contact phase and remained constant over time during the physical introduction step, as males habituated to the social situation. This pattern suggests a stabilization of relationships followed by an initial period of social exploration. Affiliation was also the most common type of social behavior observed in the wild (72.5%) and in all-male groups of Asian elephants housed in zoos (68.5%), indicating that the study animals exhibited behavior consistent with other populations [56]. The low rates of aggressive and submissive behaviors observed suggest that elephants maintained relatively stable interactions throughout the introduction process. These findings closely align with those described by Burks et al. [23] for female elephants, indicating that sequential introduction methods can successfully manage aggression while forming unrelated elephant groups. They also indicate that male elephants can form social bonds even in artificial social settings. Schreier et al. [26] reported that the proportion of aggressive behaviors was initially high during a 5-month introduction period for male zoo Asian elephants but diminished and became more affiliative over time. In this study, contrary to expectations, none of the males exhibited aggressive behaviors during the first days of introduction. As the process progressed, each male began displaying aggressive behaviors at different times and rates, revealing individual variation. The study revealed distinct behavioral patterns for the affiliative category. Four individuals—PKS, XY, BP, and JB—exhibited an increase in affiliative behaviors over time during the full physical introduction phase. Notably, PKS, characterized by its mahout as “shy” and intimidated by other males, required additional time to develop comfort within the social group, demonstrating increased affiliative behaviors later than its counterparts. He was also a juvenile and demonstrated significantly lower fAM concentrations following social interaction periods. Previous studies have reported that high-ranking musth males can suppress musth in lower-ranking males [21,57,58]. Musth, a phenomenon unique to African and Asian elephants, is typically observed in sexually mature males in good physical condition [22,59] and is associated with increased androgen production [60,61]. Although none of the male elephants in this study were in musth, the results suggest that submissive males may exhibit testosterone suppression when close to more dominant males, even outside of musth periods. JB and BP, considered shy and nondominant, also exhibited increased rates of affiliative behavior over time, although the trends were not statistically significant. By contrast, XY, labeled as an “aggressive male” by its mahout, displayed high rates of affiliative behavior, especially in the first days of introduction, which was contrary to expectations. This variation in individual responses highlights the complexity of elephant social behavior and underscores the importance of considering individual temperament when managing introductions. Research across various zoo species has demonstrated that animals exhibit diverse behavioral traits that influence their responses to management practices [62,63]. Consequently, future elephant introductions should adopt a personalized approach, accounting for individual personalities rather than applying a “one size fits all” strategy.

The observed negative effect of fAM concentrations before social interactions on fGCM could be explained by a dual-hormone regulation through cortisol’s associations with stress and social dominance [64–66]. Higher fAM may be positively related to dominance when cortisol is low, as that allows for the overt expression of dominant behaviors. Conversely, when cortisol is high, higher testosterone may actually decrease dominance and motivate lower status-seeking behavior [67]. Given the growing body of literature on the context-dependent effects of hormones, future research should continue to measure multiple hormones in conjunction with behavior to better understand these complex interactions.

In the logistic regression model, age emerged as a significant predictor of increased fAM during the full contact period. This finding suggests that older male elephants were more likely to experience elevated androgen levels following social interactions than their younger counterparts. In the context of male elephant society, age is often correlated with social status, with older males generally occupying more dominant positions in the hierarchy [21,68]. However, it is important to note that dominance status in male elephants is not static and can fluctuate over time, particularly in relation to musth [69]. This dynamic nature of elephant social hierarchies adds complexity to their social interactions and hormonal responses.

Maintaining a high-status position within such a fluid social structure requires heightened vigilance towards potential threats, particularly those that might challenge an individual’s social standing [70]. Testosterone plays a crucial role in these processes, influencing a wide range of behaviors and physiological responses. Specifically, elevated testosterone levels are associated with increased motivational drive, reduced fearfulness, and enhanced stress resilience [36,67]. In elephant society, these hormonal dynamics may help explain why older, typically more dominant males show increased androgen responses following social interactions. This heightened hormonal reactivity may be a physiological mechanism that supports their ability to gain or maintain high-status positions within the dynamic social hierarchy of male elephants [60,71].

When comparing fGCM concentrations before and after social introductions, no significant differences were found for any of the males. However, a closer examination of before-after data revealed the influence of covariates on changes in endocrine concentrations following interaction days. The fewer days since an introduction, the more likely males were to have higher fGCM concentrations following interactions during the limited contact period. This finding is consistent with the notion that during the initial exploration period, interactions may have elicited a stress response in males, as observed in other studies [26,35].

During the limited tactile contact phase, an increase in fGCM concentrations was positively predicted by the number of aggressive behaviors and negatively predicted by the number of days since first introduction. These findings suggest that aggressive behaviors may trigger an adrenal response during this period, which then diminishes over time. During the limited tactile contact period, elephants began to familiarize themselves with one another and establish a new social dominance hierarchy—a process that can be inherently stressful for the individuals involved. Previous studies in non-human primates have shown that group formation triggers the HPA axis, with glucocorticoid concentrations typically normalizing once social hierarchies stabilize [72]. A similar HPA axis response likely occurred in the ECC elephants during group formation and hierarchy establishment, which could explain why fGCM increases were negatively associated with time as social dynamics stabilized. The decrease of fGCM concentrations over time predicted by the model could also be related to the introduction process, as mahouts remained close to the males during the initial days to quickly mitigate any perceived aggressions. Mahouts stayed further away from the males as the introductions progressed over time. The close presence of the mahouts could have potentially triggered certain stress responses in the elephants during the initial days of the introduction, responses that decreased over time as the mahouts increased their observation distance. Numerous factors can influence glucocorticoid concentrations. While aversive or stressful events often trigger elevated levels, increases can also occur in response to neutral or even positive stimuli. Since rates of aggressive behavior were performed at a higher rate than aggressive or submissive behavior over time during the full physical introduction step, the increased effect observed over time on fGCM could be related to engaging in a social group. This fits with the hypothesis of a cortisol “boost,” suggesting that cortisol levels can temporarily rise during stimulating and rewarding social experiences, not just in response to stress. The increase may serve an adaptive function by enhancing alertness, engagement, and social bonding, possibly due to direct effects on metabolic processes, such as increased blood glucose levels [73]. However, further investigation is needed to support this hypothesis in Asian elephants. Another explanation for the increase in fGCM concentrations during the full contact phase may be related to the change in group composition following XY’s departure from the ECC in May 2021 and TK’s death in April 2022. The change in group composition and the re-established relationships after the departure of these males could have led to an increase in fGCM. Wild elephants have been observed showing awareness and interest in dying and deceased conspecifics [74,75], suggesting that the death of conspecifics, in addition to disrupting social groups, can be emotionally distressing for individual elephants, at least in the short term. Similarly, disruption in group composition from poaching in wild female African elephants has been associated with increased fGCM concentrations [76]. Transportation and relocation, which often result in the dissolution of social bonds or require the formation of new ones, can also trigger increased glucocorticoid excretion in elephants [77,78]. Therefore, further studies that help to understand the impact of life events on perceived stressors would be beneficial in determining the welfare needs of individuals and groups. Captive elephants could play a crucial role in the long-term preservation of the species through reintroduction efforts, breeding programs, and research initiatives. To effectively support future conservation strategies, it is crucial that the remaining captive elephant populations are managed according to the highest welfare standards.

## Conclusions

This study provides valuable insights into the social behavior and physiological responses of male Asian elephants during group formation. The prevalence of affiliative behaviors over time and the absence of significant stress responses suggest that, with proper management, all-male groups can be a viable option for elephant conservation and captive management programs. The success of the two-phase introduction process, involving limited tactile contact followed by physical introduction, indicates that a gradual approach can effectively promote positive social interactions and manage aggression during the introduction of male captive Asian elephants. Behavioral data collection proved to be a crucial tool in decision-making, allowing animal managers to obtain an instantaneous and objective assessment of each male’s behavior. Notably, affiliative behavior was more indicative of overall behavior patterns than expected, highlighting the importance of measuring these behaviors during introductions rather than focusing solely on aggressive interactions. The non-significant differences in hormone concentrations observed before and after social interactions indicate that, when properly managed, the introduction process may not be overly stressful for the elephants. However, it is crucial to note that these recommendations must be carefully tailored to each facility, taking into account available space, individual elephant temperaments, staff proficiency in interpreting behavior, and the time required to adequately monitor the introductions. Our findings, although informative, are based on a specific context, which involved managing elephants in a free-contact facility with expansive spaces that allow animals to avoid one another as needed. Therefore, conditions at the ECC may not fully capture the diversity of experiences across different facilities. This highlights the need for further research, particularly studies that examine the dynamics of all-male social groups in various settings. By questioning traditional assumptions and providing evidence-based recommendations for group formation, our study contributes to the ongoing development of best practices in elephant conservation and captive management, while also highlighting the need for continued investigation in this area. As our understanding of the social needs and behaviors of male elephants grows, we will be better equipped to design living environments and social structures that truly enhance their well-being and quality of life.

## Supporting information

S1 Fig ECC map. (PDF)

S2 Table Descriptive statistics. (DOCX)

S3 Table Wilcoxon Signed-Rank Test Summary (fGCM). (DOCX)

S4 Table Wilcoxon Signed-Rank Test Summary (fAM). (DOCX)

S5 Fig Diagnostic plots generated by the DHARMa package for fGCM models. (DOCX)

S6 Fig Diagnostic plots generated by the DHARMa package for fAM models. (DOCX)

S1 File Data set. (XLSX)
