# Supplementary figures and images for: Socializing a group of male Asian elephants in a semi-captive facility in Lao PDR
Source: PLoS One. 2025 Nov 26;20(11):e0332944. doi: 10.1371/journal.pone.0332944 (PMC12654932; doi:10.1371/journal.pone.0332944)

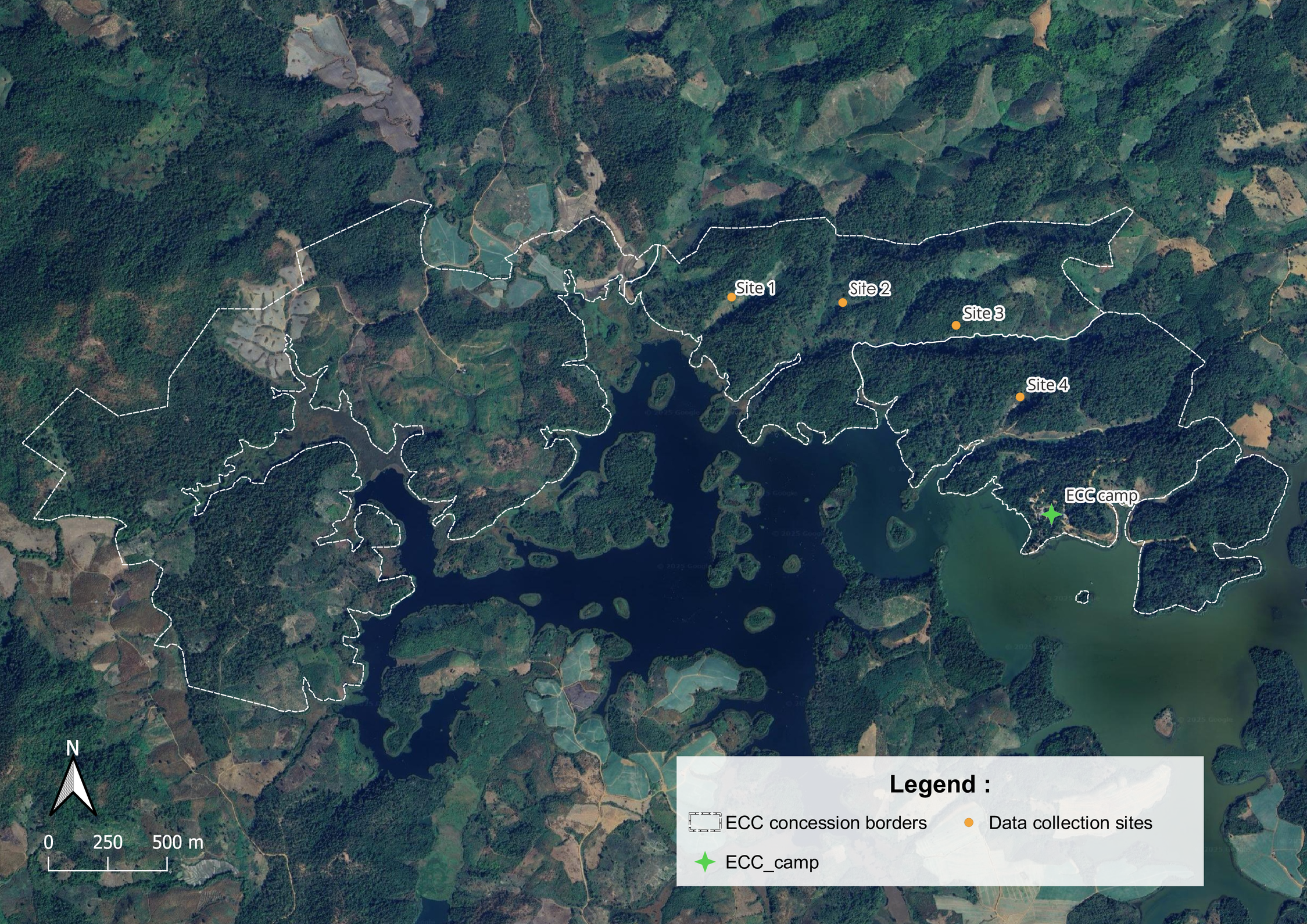

Supplement: S1 Fig — (PDF) [file pone.0332944.s001.tif]
