# Supplementary material for: Socializing a group of male Asian elephants in a semi-captive facility in Lao PDR
Source: PLoS One. 2025 Nov 26;20(11):e0332944. doi: 10.1371/journal.pone.0332944 (PMC12654932; doi:10.1371/journal.pone.0332944)
Supplement: S2 Table — (DOCX) [file pone.0332944.s002.docx]

**S2 Table**. **Descriptive statistics.** Mean (±SEM) and range (min-max) values for fecal androgen and fecal glucocorticoid concentrations before and 48 hours after social interactions for each Asian male elephant (n=8).

|  | **Male ID** | | | | | | | |
| --- | --- | --- | --- | --- | --- | --- | --- | --- |
|  | **TK** | **BB** | **XY** | **JB** | **BP** | **PKS** | **DKS** | **S** |
| **fAM before (ng/g)** | 118.05±6.92 | 134.33±6.09 | 117.92±8.04 | 101.04±5.09 | 137.23±10.56 | 171.65±9.74 | 140.95±6.31 | 108.79±5.87 |
|  | (54.24-319.49) | (39.60-347.30) | (46.51-181.58) | (12.66-284.05) | (46.07-704.56) | (52.76-550.23) | (30.77-328.31) | (28.32-315.30) |
| **fAM after (ng/g)** | 117.93±6.51 | 130.95±6.61 | 146.38±16.65 | 118.01±6.69 | 150.94±16.00 | 145.21±7.12 | 129.86±6.39 | 96.04±5.98 |
|  | (51.85-288.90) | (35.80-375.90) | (57.38-400.43) | (32.23-394.98) | (43.22-886.66) | (44.49-353.76) | (49.09-347.17) | (5.08-243.82) |
| **fGCM before (ng/g)** | 87.25±10.73 | 67.18±3.63 | 59.17±3.52 | 60.18±2.23 | 57.54±3.54 | 75.33±3.29 | 75.21±5.21 | 73.79±4.80 |
|  | (29.08-535.22) | (12.13-142.32) | (35.15-93.44) | (24.93-133.60) | (25.41-201.75) | (21.37-151.04) | (19.99-368.26) | (28.23-341.69) |
| **fGCM after (ng/g)** | 87.36±6.14 | 63.99±3.46 | 54.40-3.37 | 62.43±2.80 | 57.04±2.55 | 72.25±4.24 | 66.92±3.08 | 70.76±3.45 |
|  | (41.02-260.4) | (15.80-146.57) | (25.06-98.93) | (21.90-171.12) | (26.88-141.27) | (24.84-269.02) | (23.58-128.60) | (28.06-214.39) |

Male ID = Abbreviation name of each male; fAM before = fecal androgen metabolite concentrations before social interactions; fAM after = fecal androgen metabolite concentrations 48 hours after social interactions; fGCM.before = fecal glucocorticoid metabolite concentrations before social interactions; fGCM.after = fecal glucocorticoid metabolite concentrations 48 hours after social interactions.
