# Supplementary material for: Socializing a group of male Asian elephants in a semi-captive facility in Lao PDR
Source: PLoS One. 2025 Nov 26;20(11):e0332944. doi: 10.1371/journal.pone.0332944 (PMC12654932; doi:10.1371/journal.pone.0332944)
Supplement: S3 Table — (DOCX) [file pone.0332944.s003.docx]

| **Male ID** | **Hormone** | **Group 1** | **Group 2** | **n1** | **n2** | **Statistics** | **p-value** |
| --- | --- | --- | --- | --- | --- | --- | --- |
| BB | fGCM | fGCM.before | fGCM.after | 60 | 60 | 929 | 0.7430 |
| BP | fGCM | fGCM.before | fGCM.after | 65 | 65 | 1022 | 0.7440 |
| DKS | fGCM | fGCM.before | fGCM.after | 72 | 72 | 1630 | 0.0766 |
| JB | fGCM | fGCM.before | fGCM.after | 81 | 81 | 1527 | 0.5310 |
| PKS | fGCM | fGCM.before | fGCM.after | 81 | 81 | 1869 | 0.2330 |
| S | fGCM | fGCM.before | fGCM.after | 68 | 68 | 1228 | 0.7390 |
| TK | fGCM | fGCM.before | fGCM.after | 50 | 50 | 547 | 0.3850 |
| XY | fGCM | fGCM.before | fGCM.after | 24 | 24 | 180 | 0.4060 |

**S3 Table****. Wilcoxon Signed-Rank** **Test Summary.** Differences in fGCM concentrations before and after social interactions in male Asian elephants (n=8).

Male ID = abbreviation name of each male; fGCM = fecal glucocorticoid metabolite; fGCM.before = fecal glucocorticoid metabolite concentrations before social interactions; fGCM.after = fecal glucocorticoid metabolite concentrations 48 hours after social interactions; n1 = number of fecal samples collected before social interactions; n2 = number of fecal samples collected 48hours after social interactions.
