# Supplementary material for: Socializing a group of male Asian elephants in a semi-captive facility in Lao PDR
Source: PLoS One. 2025 Nov 26;20(11):e0332944. doi: 10.1371/journal.pone.0332944 (PMC12654932; doi:10.1371/journal.pone.0332944)
Supplement: S4 Table — (DOCX) [file pone.0332944.s004.docx]

| **Male ID** | **Hormone** | **Group 1** | **Group 2** | **n1** | **n2** | **Statistic** | **p-value** |
| --- | --- | --- | --- | --- | --- | --- | --- |
| BB | fAM | fAM.before | fAM.after | 60 | 60 | 956.5 | 0.7630 |
| BP | fAM | fAM.before | fAM.after | 63 | 63 | 949.0 | 0.6890 |
| DKS | fAM | fAM.before | fAM.after | 72 | 72 | 1606.0 | 0.1020 |
| JB | fAM | fAM.before | fAM.after | 76 | 76 | 1088.0 | 0.0525 |
| PKS | fAM | fAM.before | fAM.after | 81 | 81 | 2125.0 | 0.0289 |
| S | fAM | fAM.before | fAM.after | 68 | 68 | 1398.0 | 0.1700 |
| TK | fAM | fAM.before | fAM.after | 50 | 50 | 549.0 | 0.5310 |
| XY | fAM | fAM.before | fAM.after | 24 | 24 | 83.0 | 0.0974 |

**S4 Table. Wilcoxon Signed-Rank Test Summary.** Differences in fAM concentrations before and 48h after social interactions in male Asian elephants (n=8).

Male ID = abbreviation name of each male; fAM = fecal androgen metabolite; fAM.before = fecal androgen metabolite concentrations before social interactions; fAM.after = fecal androgen metabolite concentrations 48 hours after social interactions; n1 = number of fecal samples collected before social interactions; n2 = number of fecal samples collected 48 hours after social interactions.
