# Supplementary material for: Socializing a group of male Asian elephants in a semi-captive facility in Lao PDR
Source: PLoS One. 2025 Nov 26;20(11):e0332944. doi: 10.1371/journal.pone.0332944 (PMC12654932; doi:10.1371/journal.pone.0332944)
Supplement: S6 Fig — (DOCX) [file pone.0332944.s006.docx]

1. **Limited Tactile Contact**

| 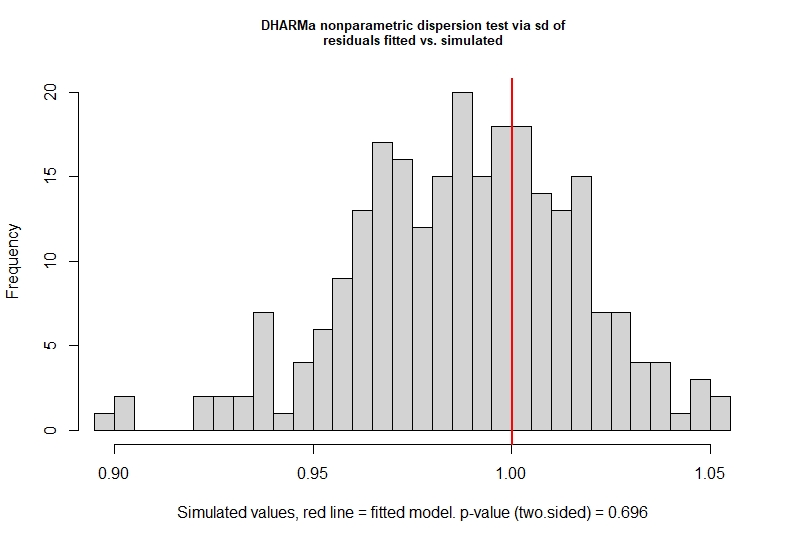 |
| --- |
| 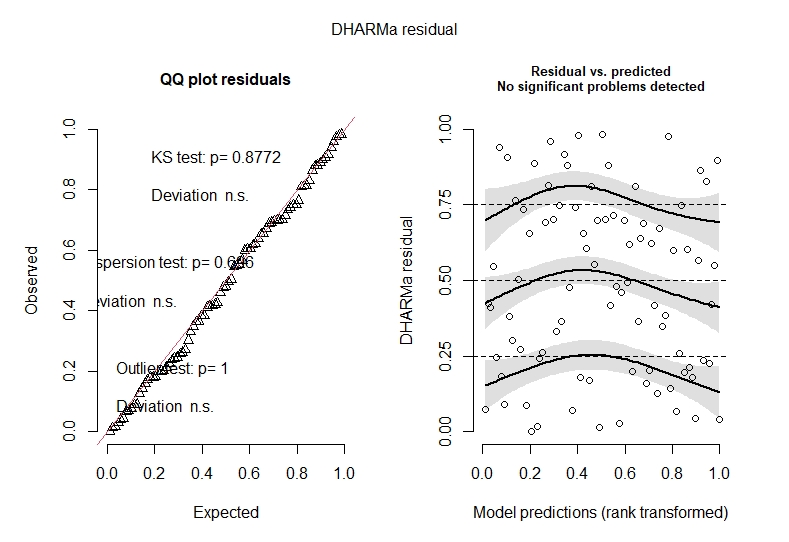 |

1. **Physical Introduction**

| 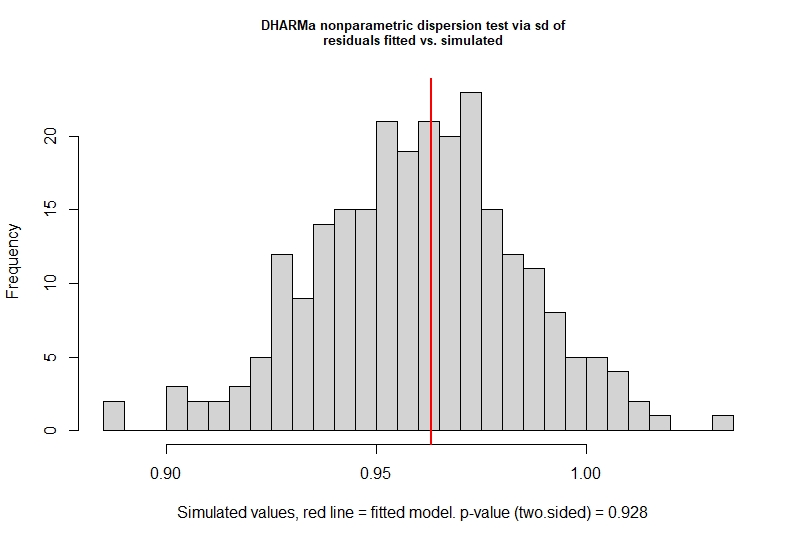 |
| --- |
| 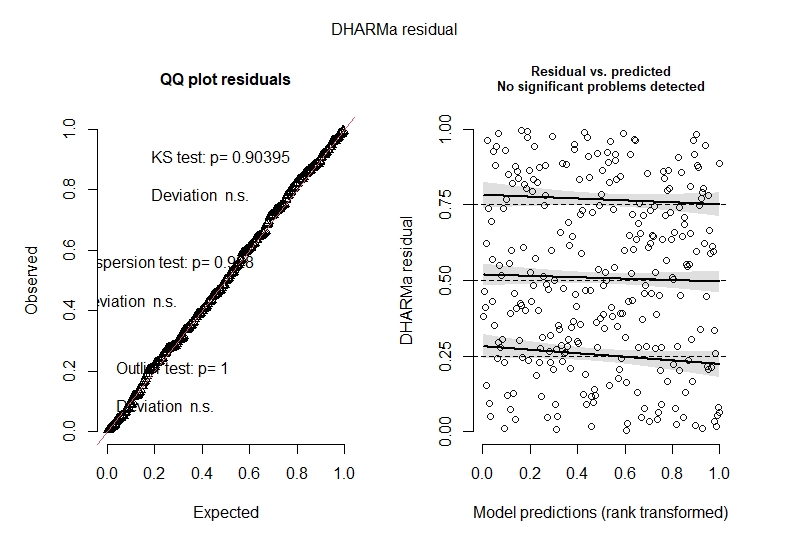 |

**S6 Fig. Diagnostic plots generated by the DHARMa package to assess the residuals of a fitted model for fecal androgen metabolite concentrations (fAM).**

a) limited tactile contact model, and b) physical introduction model. Plots display quantile-quantile (QQ) distribution, residuals vs fitted values, and outlier detection to assess model validity.
